# Automatic radiotherapy planning for deliverable plans using deep learning dose prediction and dose rings optimization in cervical cancer

**DOI:** 10.1002/acm2.70353

**Published:** 2025-11-12

**Authors:** Weiqian Huang, Ting Liu, Yichao Shen, Ziqing Xiang, Dong Wang, Wen Fu, Li Shao, Xianwen Yu, Weihua Ni, Yongqiang Zhou, Huan Liu, Ce Han, Xiance Jin, Ji Zhang

**Affiliations:** ^1^ Department of Radiotherapy and Medical Oncology 1st Affiliated Hospital of Wenzhou Medical University Wenzhou China; ^2^ The 1^st^ School of Medicine (School of information and Engineering) Wenzhou Medical University Zhejiang China; ^3^ Department of Radiation Oncology Taizhou Hospital Taizhou China; ^4^ Department of Medical Engineering and Equipment 2nd Affiliated Hospital of Wenzhou Medical University Wenzhou China; ^5^ Cixi Biomedical Research Institute Wenzhou Medical University Zhejiang China; ^6^ School of Basic Medical Science Wenzhou Medical University Wenzhou China

**Keywords:** automatic planning, deep learning, dose rings optimization, intensity‐modulated radiation therapy, volumetric modulated arc therapy

## Abstract

**Background:**

Automatic radiotherapy (RT) planning based on deep learning (DL) has been extensively researched. However, it is challenging to import the predicted dose distribution into mainstream treatment planning systems (TPSs) and generate clinically deliverable plans.

**Purpose:**

To investigate the feasibility and accuracy of an automatic volumetric modulated arc therapy (VMAT) and intensity‐modulated radiation therapy (IMRT) planning method for generation of universally deliverable plans based on DL dose prediction and dose rings optimization.

**Methods:**

First, dose distributions were predicted using a three‐dimensional (3D) Fusion Residual Unet (F‐ResUnet) DL network with data from two hospitals, which included 230 and 210 gynecological cancer (GC) patients underwent VMAT and IMRT, respectively. Then, the predicted dose distributions were discretized into dose rings to optimize the plans automatically in two mainstream TPSs based on the dose rings. Finally, the deliverability of generated plans was verified with patient‐specific quality assurance (PSQA).

**Results:**

The predicted dose distributions were clinically acceptable with a target coverage over 95%. Compared with the clinical plans, the automatic plans optimized with dose rings achieved a similar dose coverage on planning target volumes (PTV) with an average target coverage over 96.5%. For organs at risk (OARs) sparing, automatic VMAT plans markedly decreased the V_30Gy_ of left femoral head (*p* = 0.05), right femoral head (*p* = 0.004), and small intestine (*p* = 0.04). The V_45Gy_ of bladder and rectum in the automatic IMRT plans were reduced by approximately 7% and 9%, respectively. Deliverability verification with PSQA achieved a mean gamma passing rate of 99.1%, 97.1% and 98.3%, 95.0% under the criteria of 3%/3 mm and 3%/2 mm for VMAT and IMRT plans, respectively.

**Conclusions:**

The proposed automatic planning method combining DL dose prediction and dose rings optimization was feasible to generate universally deliverable VMAT and IMRT plans for gynecological cancer (GC) patients.

## 1 INTRODUCTION

With the advancements of computer science and medical imaging, radiotherapy (RT) has progressed enormously from traditional two‐dimensional (2D) RT to three‐dimensional (3D) conformal radiotherapy (CRT), and to precise RT technologies of and intensity‐modulated radiation therapy (IMRT) and volumetric modulated arc therapy (VMAT).^1^, ^2^ Although, the advanced RT technologies improved the RT plan quality and clinical outcomes, they also increased the treatment planning time and plan quality variability resulted from the iterative and meticulous trial‐and‐error fashion and back‐and‐forth interaction between physicians and planners during planning.^3^ Automatic planning method with knowledge‐based planning (KBP) was initially developed to decrease the treatment planning time and to improve the consistency of plan quality.^4^, ^5^ In which, features were extracted from contoured structures of previously treated patients to generate dose‐volume histogram (DVH) objectives for a new patient.^6^ However, one key limitation of DVH based KBP was the lack of spatial information.^7^, ^8^ On the other hand, KBP still requires lots of human manipulation when using the extracted anatomical features to improve the plan quality.^9^, ^10^

With the advances of deep learning (DL), convolutional neural network (CNN) based algorithms were extensively investigated to predict the dose distributions directly by extracting nonlinear higher‐order features from target volumes and organs at risk (OARs) for RT planning.^11^, ^12^, ^13^ However, studies pointed out that it was challenging to convert predicted 3D spatial dose distributions from CNN models into clinically deliverable plans due to the technical complexities in the training network.^14^ Predicted dose distributions from CNNs may be irreproducible and not readily applicable in a real clinic condition.^15^, ^16^, ^17^ The studies of Fan et al. and Shen et al. focused on generating voxel‐based plans in open‐source treatment planning system (TPS), which is not suitable for real‐world treatment delivery.^18^, ^19^ Zhong et al. and Sun et al. developed automatic RT planning strategy based on dose mimic in specific UIH TPS (United Imaging Healthcare, Shanghai, China), which had a small scope of application.^20^, ^21^ Studies based on DL to predict multi‐leaf collimator (MLC) motion sequences and fluence maps of IMRT or VMAT plans for direct treatment plan generation have also been reported.^22^, ^23^, ^24^ The plans predicted by fluence map were usually unoptimized with still room for improvement and had a low flexibility in plan adjustment.

To address these research gaps, a universally applicable and deliverable automatic planning method was proposed by integrating DL dose prediction and dose rings optimization in this study. First, dose distributions of IMRT and VMAT plans were predicted using DL with data from two hospitals. Then, the dose distributions were discretized into dose rings and automatically optimized within two mainstream TPSs (Monaco 5.1.1; Elekta Ltd, Crawley, UK and Pinnacle 9.0, Philips Medical Systems, Inc., Fitchburg, WI, USA) based on the dose ring structures. Finally, the plan deliverability was verified with measurement‐based patient‐specific quality assurance (PSQA). This suggested method is promising to overcome the limitations of current automatic planning methods relayed on TPSs and to be universally applicable and deliverable in different mainstream commercial TPSs.

## 2 MATERIALS AND METHODS

### 2.1 Patients and plans

Patients with gynecologic cancers (GC) treated by VMAT in hospital one and treated by IMRT in hospital two from year 2021 to 2023 were retrospectively enrolled and analyzed in this study. The VMAT plans were generated using Monaco TPS with two arcs and two different prescription doses: one was 45 Gy in 25 fractions for single‐target cases, and the second was 45 and 50 Gy in 25 fractions for simultaneous integrated boost (SIB) cases. IMRT plans were generated using Eclipse TPS (version 10.0, Varian Medical Systems, Palo Alto, CA, USA) with seven fields (beam angles at 40°, 100°, 155°, 180°, 205°, 260°, and 320°) at a prescription dose of 45 Gy in 25 fractions for single‐target cases. The VMAT and IMRT datasets were randomly divided into training sets of 188 and 190 cases, validation sets of 20 and 10 cases, and test sets of 22 and 10 cases, respectively. Models were constructed for VMAT datasets and IMRT datasets respectively. The OARs included rectum, bladder, small intestine, and left and right femoral head. The enrolled plans were renormalized to ensure that the planning target volume (PTV) was covered by at least 95% of the prescription dose. The hospital's institutional review board reviewed and approved this study (ECCR No. 2019059) with consent requirement was waived due to retrospective and simulation nature of the study.

### 2.2 Data preprocessing and dose prediction model

The DICOM processing package Pydicom and scientific computing package Numpy were used to preprocess 3D images of computed tomography (CT), PTVs, OARs and dose distributions.^25^, ^26^ The CT images were first adjusted by a window width of 600 and level of 40, then the voxels within the –1000–000 HU were normalized to a range of [0, 1] for dose prediction after data augmentation.^27^ All images were interpolated to a resolution of 3 mm × 3 mm × 3 mm, with a normalized maximum dose of 55 Gy for VMAT and 50 Gy for IMRT.^28^ The inputs of the model included three channels: CT image slices, PTV contours (PTV45 and PTV50), and OAR contours, while the outputs were the corresponding 3D dose distributions.

**FIGURE 1:**
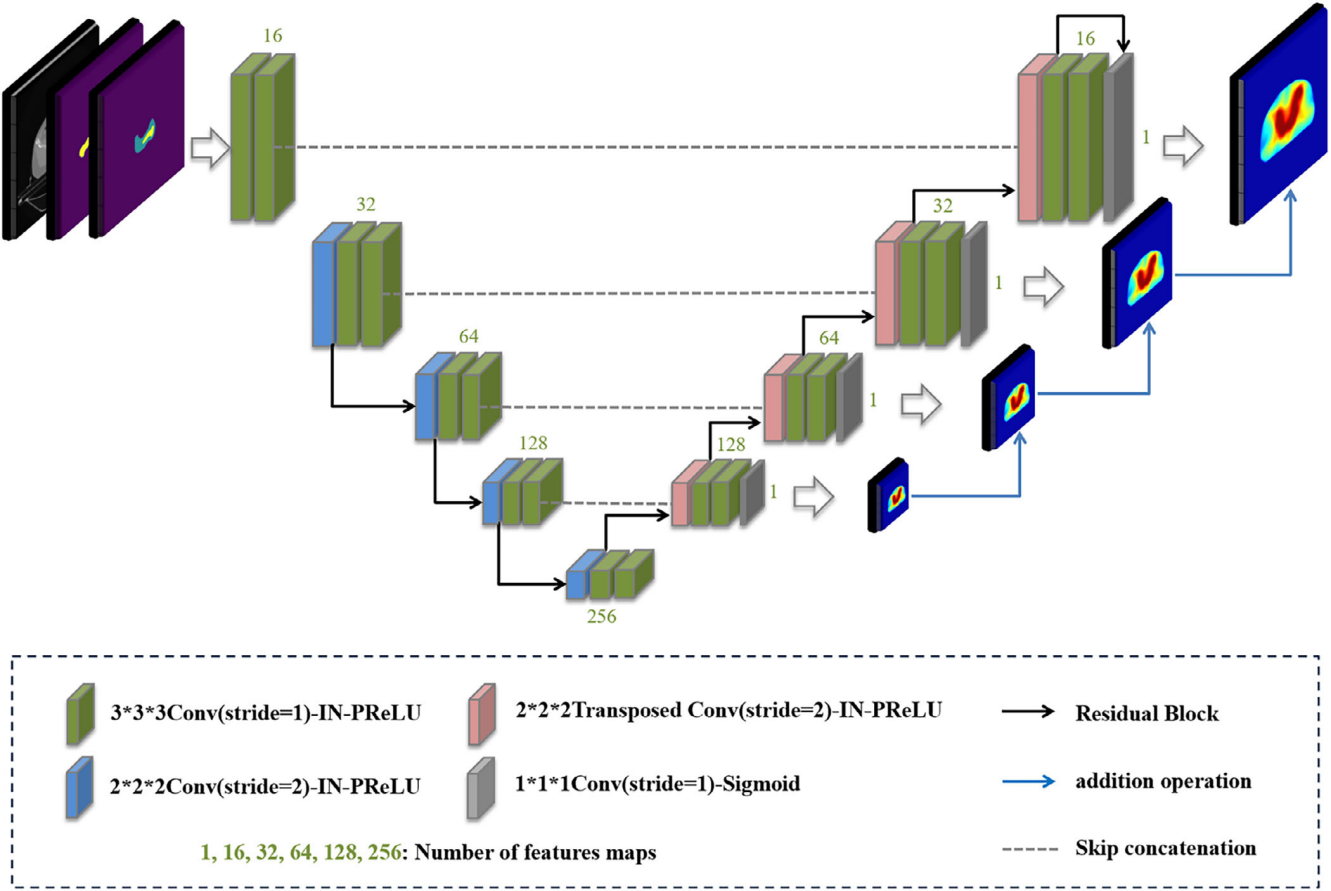
Architecture of the 3D F‐ResUnet used for dose prediction.

A 3D Fusion Residual Unet (F‐ResUnet) with U‐net‐like structure,^29^ which is able to fuse information from the lower‐level network, was adopted to predict the dose distribution, as shown in Figure 1. The down‐sampling path and up‐sampling path consisted of similar modules: 2 × 2 × 2 convolutions layers or transposed convolutions layers, instance normalization (IN) layers, and parametric rectified linear units (PReLUs). Residual blocks were interspersed within the encoder and decoder modules of the model. In the decoder module of the network, a prediction result was generated after each up‐sampling and 1 × 1 × 1 convolution layer to obtain the dose distribution at different resolutions, then the low‐level prediction results were up‐sampled by a factor of 2 and added to the high‐level prediction results to convey more image information.^30^ Thus, a more accurate dose distribution with rich image details was generated.

This network was used to train two models for the VMAT datasets and the IMRT datasets respectively. Mean squared error (MSE), mean absolute error (MAE), and Rank loss^30^ were applied for the model training. The Adam optimization algorithm was used to minimize the loss function value between the predicted dose and the clinical truth with an initial learning rate of 0.0001. An NVIDIA GeForce GTX 3090 graphical processing unit was applied for the model training. The training details and model comparisons were presented in the Supporting Information.

### 2.3 Isodose line regions generation

**FIGURE 2:**
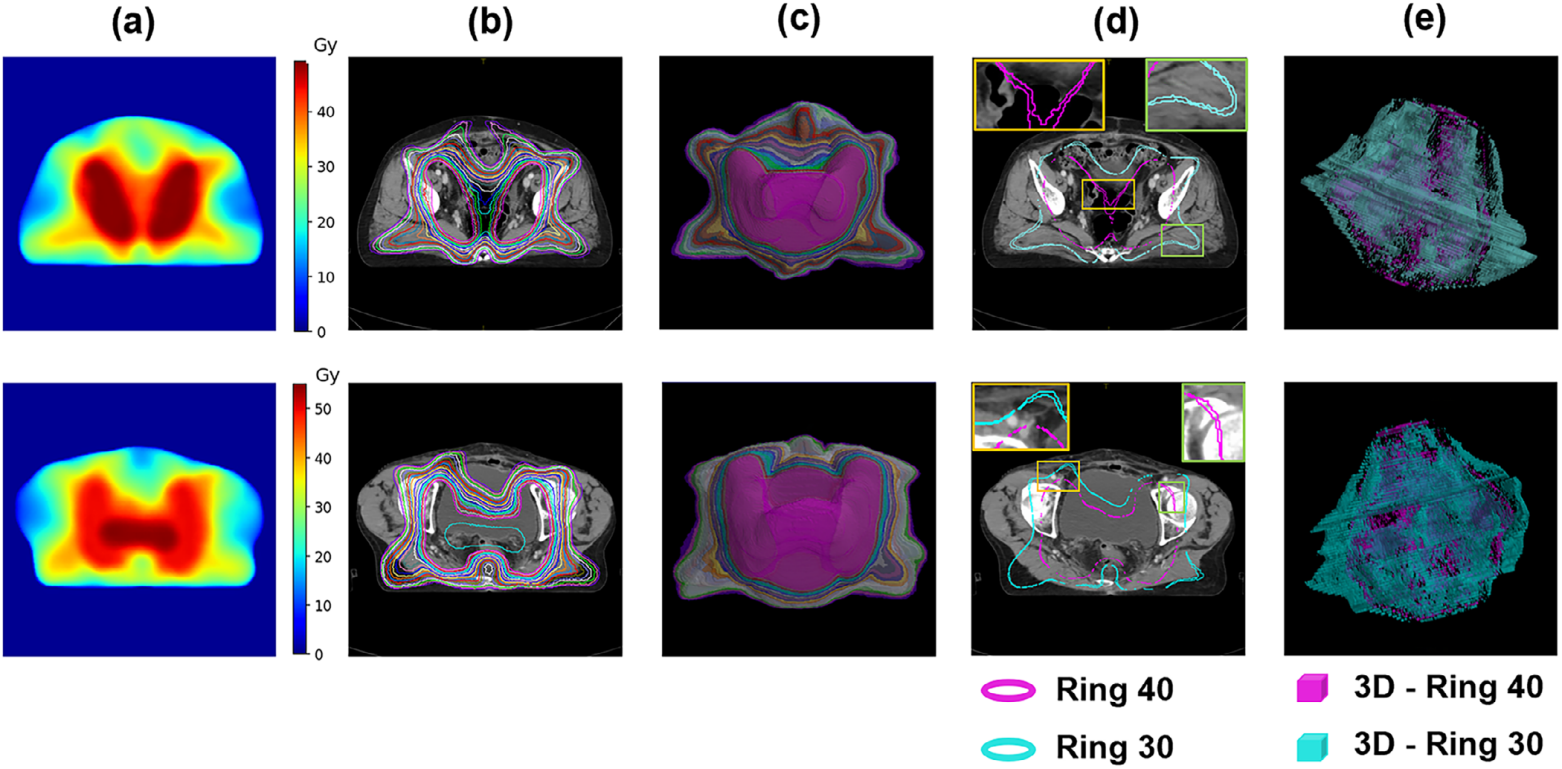
Predicted dose distributions and corresponding Isodose line regions (ISO regions) and ring structures. (a) Predicted dose distribution. (b) Transverse sections of ISO regions with a dose level greater than 25 Gy (different colors only represent different dose levels). (c) 3D structures corresponding to the ISO regions in (b). (d) Transverse sections of Ring 40 and Ring 30. The boxes were the local zoomed‐in views. (e) 3D structures of Ring 40 and Ring 30.

The dose distributions predicted by F‐ResUnet were processed using Python to generate matrices of discrete isodose line regions (ISO regions), as shown in Figure 2b,c. During the generation of ISO regions, a specific algorithm was devised to discretize the dose distribution: multiple ISO regions were composed of dose levels ranging from 1 to *n* at an interval of 1 Gy, where *n* represents the prescription dose. For an individual ISO region, all voxel values in the matrix that exceeded the current dose level were set to 255 (indicating inside the ISO region), while all other voxel values were set to 0 (indicating outside the ISO region). Figure 2b showed the transverse sections of ISO regions with a dose level greater than 25 Gy, with different colors only represented different dose levels. The 3D structures in Figure 2c allowed for a clear observation of continuous ISO regions. Subsequently, the n ISO regions were converted into n regions of interest (ROIs) using the Python package RTutils and integrated into a single RTStruct file, which was then imported into the TPS for further optimization.

### 2.4 Automatic optimization based on dose rings

The subtraction of two adjacent ISO regions can produce a dose ring region. By sequentially subtracting the (n – 1)th ISO region from the nth ISO region, a series of (n – 1) dose ring regions can be obtained, denoted as Ring 1, Ring 2, up to Ring (*n* – 1), corresponding to dose ranges from 1–2 Gy, 2–3 Gy, up to (*n* – 1)—n Gy, respectively. Ring n corresponded to the nth ISO region. To enhance the clarity of visualizing the Ring structure, only the transverse sections of Ring 30 and Ring 40 were shown in Figure 2d, and Figure 2e showed the 3D structures corresponding to the dose ring. Subsequently, constraint functions were set for each Ring to facilitate the generation of treatment plans. Considering that imposing constraints on an excessive number of rings would reduce the planning efficiency, unnecessary low‐dose constraints below Ring 25 were excluded.

Table 1 showed the dose ring constraint functions for VMAT plans of two cases, single‐target and SIB. Rings outside the target area were assigned an identical weight when using Monaco TPS for VMAT optimization to approximate the dose distributions predicted by the DL model. IMRT plans were optimized with Pinnacle TPS with similar dose ring constrain functions but emphasized on the intersection between OARs and rings in order to reduce the dose in these intersection regions, as shown in Table 2. Plans were automatically generated for 22 VMAT patients and 10 IMRT patients in the test set using the constraint functions without any adjustments. The efficiency and computation time for the suggested optimization method were investigated and recorded.

**TABLE 1:** Dose ring constraint functions for VMAT plans in Monaco treatment planning system.

		Single‐target patients			SIB patients		
Structure	Cost function	Weight	Reference dose(cGy)	Isoconstraint	Weight	Reference dose(cGy)	Isoconstraint
Ring 50	Target penalty	–	–	–	1.00	–	5000.0
	Underdose DVH	–	–	–	100.00	5000.0	96.50
	Maximum dose	–		–	10.00	–	5500.0
Ring 45	Target penalty	10	–	4500	3.00	–	4500.0
	Underdose DVH	–	–	–	100.00	4500.0	98.50
	Maximum dose	–	–	–	1.00	–	5000.0
Ring 44	Overdose DVH	0.01	4500.0	0.01	0.01	4500.0	0.01
Ring 43	Overdose DVH	0.01	4400.0	0.01	0.01	4400.0	0.01
Ring 42 … … Ring 25	Overdose DVH … … Overdose DVH	0.01 … … 0.01	4300.0 … … 2600.0	0.01 … … 0.01	0.01 … … 0.01	4300.0 … … 2600.0	0.01 … … 0.01
Body	Maximum dose	1	–	5000	1.00	–	5500.0

Abbreviations: DVH, dose‐volume histogram; SIB, simultaneous integrated boost.

**TABLE 2:** Dose ring constraint functions for the IMRT optimization plans in Pinnacle treatment planning system.

Structure	Cost function	Weight	Reference dose(cGy)
Ring 45	Min dose	100.00	4500.0
	Max dose	80.00	4650.0
	Min dvh	100.00	4500/100%
	Uniform dose	10.00	4550.0
Ring 44	Max dose	0.01	4500.0
Ring 43	Max dose	0.01	4400.0
Ring 42 … … Ring 25	Max dose … … Max dose	0.01 … … 0.01	4300.0 … … 2600.0
Ring 30 ∩ organ	Max dose	0.01	3000.0
Ring 33 ∩ organ	Max dose	0.01	3300.0
Ring 36 ∩ organ	Max dose	0.01	3600.0
Ring 39 ∩ organ	Max dose	0.01	3900.0

Abbreviations: ∩, the intersection between organ and ring; dvh, dose‐volume histogram.

### 2.5 Quality assurance of automatic plan

The deliverability of generated VMAT and IMRT plans was verified through the Elekta Infinity (Elekta Ltd, Crawley, UK) and Varian Trilogy (Varian Medical Systems, Palo Alto, CA, USA) linear accelerators, respectively. This verification was achieved by employing standard PSQA with ArcCHECK (Sun Nuclear Corp., Melbourne, FL, USA) under the gamma passing criteria of 3 %/3 mm and 3%/2 mm.^31^, ^32^ The purpose of this PSQA implementation was to comprehensively assess the feasibility and accuracy of automatically generated VMAT and IMRT plans from the perspective of actual treatment delivery.

### 2.6 Evaluation metrics and statistical analysis

The performance of dose prediction models and the accuracy of dose ring‐based optimization were evaluated by comparing the predicted dose distribution and automatically optimized results with the dose distributions of clinically accepted plans. Dosimetric parameters of PTV (*V*_45Gy_, *V*_50Gy_, *D*_95,_ homogeneity index (HI), and conformity index (CI)), and OARs (*V*_45Gy_, *V*_40Gy_, *V*_30Gy_, *V*_20Gy_, and *D*_mean_), as well as isodose line similarity by dice similarity coefficient (DSC) were applied for evaluation.^33^, ^34^, ^35^ Detailed definition of these evaluation metrics was presented in Supporting Information. All dosimetric comparisons were analyzed using the paired *t*‐test with a significance level of 0.05.

## 3 RESULTS

### 3.1 Patients

A total of 230 VMAT and 210 IMRT patients were enrolled in this study from hospital one and hospital two, respectively. There were 172 cervical cancer (CC) and 58 endometrial cancer patients in VMAT group with a mean age of 55 (range, 38−79) years. All the cases in IMRT group were CC patients with a mean age of 57 (range, 29−84) years. The average PTV volume of VMAT and IMRT patients was 1098 and 1447 cm^3^, respectively. Detailed clinical characteristics of enrolled patients was shown in Table 3.

**TABLE 3:** Detailed clinical characteristics of enrolled patients.

	VMAT patients		IMRT patients
Characteristic	Single‐target cases	SIB cases	
Dataset (numbers)	Training (142) Validation (13) Testing (14)	Training (46) Validation (7) Testing (8)	Training (190) Validation (10) Testing (10)
Average age (range)	55 (38−79)		57 (29−84)
Cancer (numbers)	Cervical cancer (118) Endometrial cancer (51)	Cervical cancer (54) Endometrial cancer (7)	Cervical cancer (all)
PTV volume (average)	PTV45 (1098 cm^3^) PTV50 (116 cm^3^)		PTV45(1447 cm^3^)

Abbreviations: IMRT, intensity‐modulated radiation therapy; PTV, planning target volume; PTV45, the target volume with a prescription dose of 45 Gy; PTV50, the target volume with a prescription dose of 50 Gy; SIB, simultaneous integrated boost; VMAT, volumetric modulated arc therapy.

### 3.2 Performance of dose prediction

**FIGURE 3:**
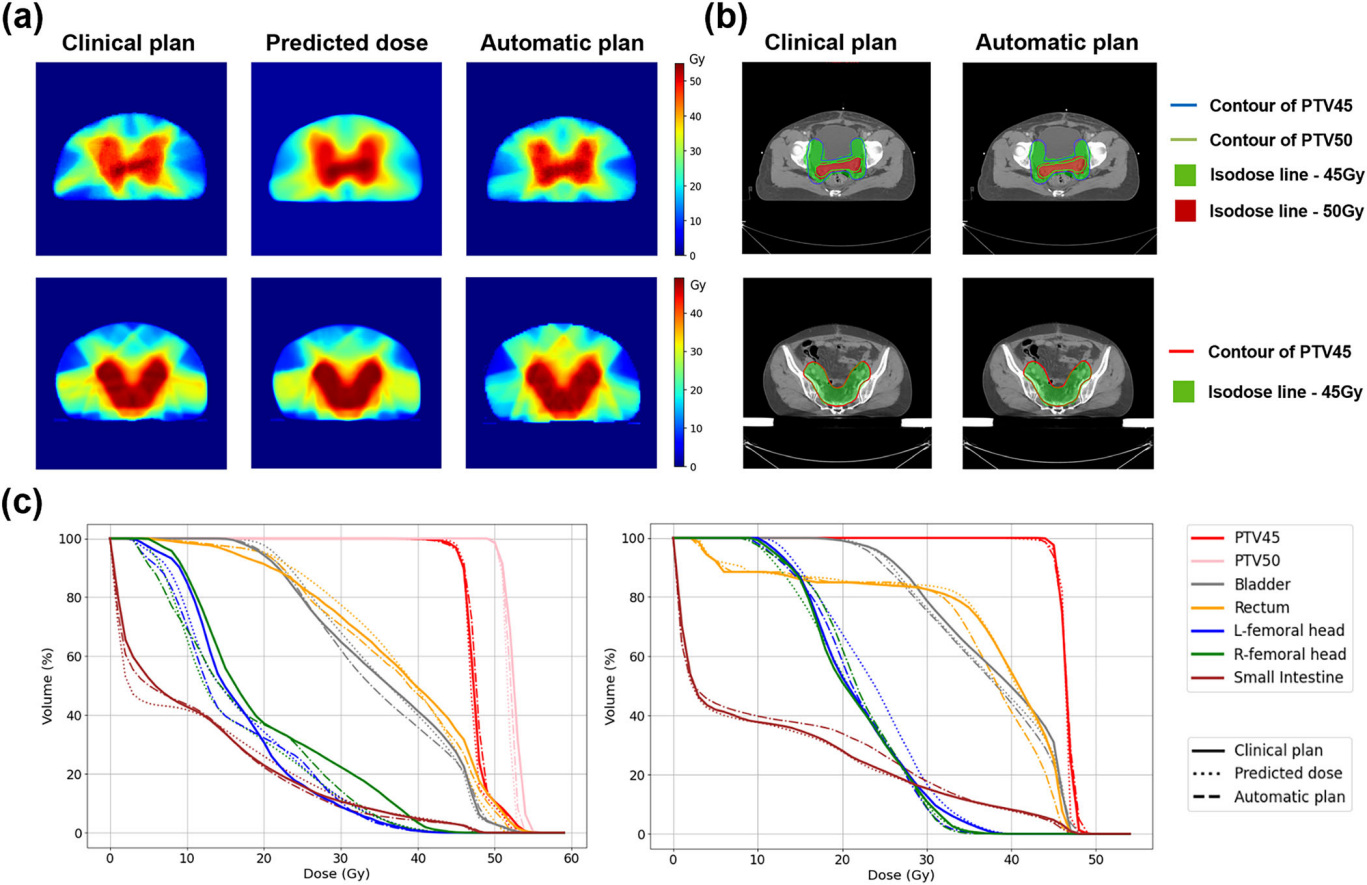
Dose comparison for two typical example test set patients. (a) The dose distribution comparison of the clinical plan, predicted dose and automatic plan. The first row was a test VMAT case. The second row was a test IMRT case. (b) The target coverage of isodose lines for clinical plan and automatic plan. (c) Dose‐volume histogram (DVH) comparison. The DVH on the left was the corresponding VMAT case and on the right was the corresponding IMRT case.

The predicted dose distributions in the target volumes for IMRT and VMAT plans were slightly higher than those of real clinical cases, but the dose distributions were still clinically acceptable with a coverage over 95%. Specifically, as illustrated in the detailed dose metrics presented in Tables 4 and 5, the predicted values of *V*_50Gy_, *V*_45Gy_, and *D*_95_ were higher in comparison to the clinical VMAT and IMRT plans. However, the predicted dose distribution improved the conformity and homogeneity of PTV. There was no significant difference on OARs between predicted and real clinical dose distributions for all VMAT and IMRT plans (P1 > 0.05). The comparisons between the predicted dose and the clinical plans in terms of DVHs and dose distributions were shown in Figure 3.

**TABLE 4:** The comparison of dose metrics for 22 VMAT patients.

Organs	Dose metrics	Clinical plans (real dose)	Predicted dose	Automatic plans	P1	P2	P3
PTV50	*V*_50Gy_(%)	96.90 ± 1.35	98.59 ± 0.75	96.97 ± 1.06	**0.01**	0.90	**0.003**
	*D*_95_(Gy)	50.63 ± 0.37	50.95 ± 0.38	50.58 ± 0.46	0.10	0.81	0.10
PTV45	*V*_45Gy_(%)	97.26 ± 1.18	99.10 ± 0.61	97.29 ± 0.78	**<0.001**	0.90	**<0.001**
	*D*_95_(Gy)	45.53 ± 0.29	46.19 ± 0.22	45.44 ± 0.22	**<0.001**	0.25	**<0.001**
	CI	0.81 ± 0.02	0.82 ± 0.02	0.82 ± 0.02	0.08	**0.03**	0.6
	HI	1.06 ± 0.01	1.03 ± 0.00	1.06 ± 0.00	**<0.001**	0.94	**<0.001**
Bladder	*D*_mean_ (Gy)	35.79 ± 2.56	36.21 ± 2.40	35.03 ± 2.62	0.57	0.34	0.13
	*V*_45Gy_(%)	28.98 ± 7.82	29.31 ± 10.04	27.53 ± 9.58	0.90	0.59	0.55
	*V*_20Gy_(%)	91.75 ± 8.73	95.12 ± 4.34	93.81 ± 3.89	0.11	0.32	0.30
Rectum	*D*_mean_(Gy)	36.59 ± 2.80	37.03 ± 2.69	37.59 ± 2.69	0.60	0.24	0.50
	*V*_45Gy_(%)	32.87 ± 10.16	33.80 ± 9.56	32.73 ± 9.7	0.76	0.96	0.71
	*V*_20Gy_(%)	88.71 ± 6.13	90.26 ± 5.74	92.41 ± 5.21	0.39	**0.04**	0.20
L‐femoral head	*D*_mean_(Gy)	16.35 ± 1.89	16.58 ± 1.72	15.95 ± 1.94	0.68	0.49	0.26
	*V*_30Gy_(%)	12.50 ± 5.42	13.82 ± 4.31	9.57 ± 3.96	0.38	0.05	**0.002**
R‐femoral head	*D*_mean_(Gy)	16.78 ± 1.85	16.26 ± 1.56	15.74 ± 1.53	0.32	0.05	0.27
	*V*_30Gy_(%)	14.31 ± 6.25	13.28 ± 4.08	9.47 ± 4.17	0.52	**0.004**	**0.004**
Small intestine	*D*_mean_(Gy)	12.18 ± 3.37	12.40 ± 3.52	11.43 ± 3.39	0.83	0.47	0.36
	*V*_40Gy_(%)	8.36 ± 4.01	8.44 ± 4.29	6.48 ± 3.39	0.95	0.10	0.10
	V_30Gy_(%)	17.64 ± 6.93	18.20 ± 7.60	13.50 ± 5.95	0.80	**0.04**	**0.03**

*Note*: Mean value and standard deviations are shown. *P1* significant difference between clinical plans and predicted dose, *P2* significant difference between clinical plans and automatic plans, *P3* significant difference between predicted dose and automatic plans. Results with*p* < 0.05 indicated statistical significance and were labeled with bold.

Abbreviations: PTV45, the target volume with a prescription dose of 45 Gy; PTV50, the target volume with a prescription dose of 50 Gy.

**TABLE 5:** The comparison of dose metrics for 10 IMRT patients.

Organs	Dose metrics	Clinical plans (real dose)	Predicted dose	Automatic plans	P1	P2	P3
PTV45	*V*_45Gy_(%)	97.38 ± 1.19	98.36 ± 0.50	96.5 ± 1.27	0.06	0.13	0.75
	*D*_95_(Gy)	45.69 ± 0.72	45.91 ± 0.08	45.15 ± 0.27	0.05	**0.04**	0.54
	CI	0.90 ± 0.10	0.93 ± 0.04	0.87 ± 0.01	**<0.001**	0.36	**<0.001**
	HI	1.06 ± 0.00	1.03 ± 0.04	1.08 ± 0.01	**<0.001**	**<0.001**	**<0.001**
Bladder	*D*_mean_ (Gy)	38.73 ± 1.92	38.14 ± 2.10	38.74 ± 1.65	0.52	1.00	0.49
	*V*_45Gy_(%)	32.36 ± 5.66	30.32 ± 6.22	25.68 ± 4.79	0.45	**0.01**	0.08
	*V*_20Gy_(%)	98.99 ± 1.91	98.95 ± 2.11	99.88 ± 0.35	0.97	0.16	0.19
Rectum	*D*_mean_(Gy)	36.31 ± 2.52	36.57 ± 2.46	35.79 ± 2.28	0.82	0.64	0.47
	*V*_45Gy_(%)	23.71 ± 11.45	26.01 ± 10.41	14.12 ± 8.04	0.64	**0.04**	**0.01**
	*V*_20Gy_(%)	87.50 ± 7.37	87.24 ± 6.81	88.17 ± 7.50	0.93	0.84	0.77
L‐femoral head	*D*_mean_(Gy)	26.93 ± 2.53	27.79 ± 2.50	25.15 ± 1.99	0.45	0.10	**0.02**
	*V*_30Gy_(%)	34.47 ± 11.1	40.21 ± 12.30	37.11 ± 20.21	0.29	0.72	0.68
R‐femoral head	*D*_mean_(Gy)	27.07 ± 2.92	26.7 ± 2.88	26.51 ± 2.40	0.78	0.65	0.87
	*V*_30Gy_(%)	37.22 ± 13.5	43.63 ± 11.97	40.91 ± 22.20	0.22	0.66	0.6
Small intestine	*D*_mean_(Gy)	11.91 ± 2.47	11.89 ± 2.40	12.45 ± 2.60	0.99	0.64	0.62
	*V*_40Gy_(%)	7.18 ± 2.13	6.98 ± 2.11	7.28 ± 2.08	0.83	0.92	0.75
	*V*_30Gy_(%)	15.01 ± 4.25	14.66 ± 3.95	17.13 ± 5.03	0.85	0.32	0.24

*Note*: Mean value and standard deviations are shown. *P1* significant difference between clinical plans and predicted dose, *P2* significant difference between clinical plans and automatic plans, *P3* significant difference between predicted dose and automatic plans. Results with *p* < 0.05 indicated statistical significance and were labeled with bold.

Abbreviations: PTV45, the target volume with a prescription dose of 45 Gy; PTV50, the target volume with a prescription dose of 50 Gy.

### 3.3 Dose rings optimization assessment

The automatic plans optimized with dose rings achieved a similar dose coverage on PTV in comparison with clinically accepted IMRT and VMAT plans. As shown in Table 4, except for CI of PTV45 (P2 = 0.03), there was no significant dose difference on target volumes between automatic VMAT plans and clinical plans. The PTV coverage of IMRT automatic plans was slightly lower than that of the clinical plans, but still reached 96.5% (Table 5). For OAR sparing, automatic VMAT plans optimized with dose rings markedly decreased the *V*_30Gy_ of left femoral head (P2 = 0.05), right femoral head (P2 = 0.004), and small intestine (P2 = 0.04), but it increased a bit of the *V*_20Gy_ of bladder (P2 = 0.32) and rectum (P2 = 0.04), in comparison with clinically accepted plans (Table 4). The IMRT automatic plans optimized with dose rings substantially reduced the *V*_45Gy_ of bladder (32.36% ± 5.66% vs. 25.68% ± 4.79%) and rectum (23.71% ± 11.45% vs. 14.12% ± 8.04%), representing reductions of approximately 7% and 9%, respectively (Table 5).

**FIGURE 4:**
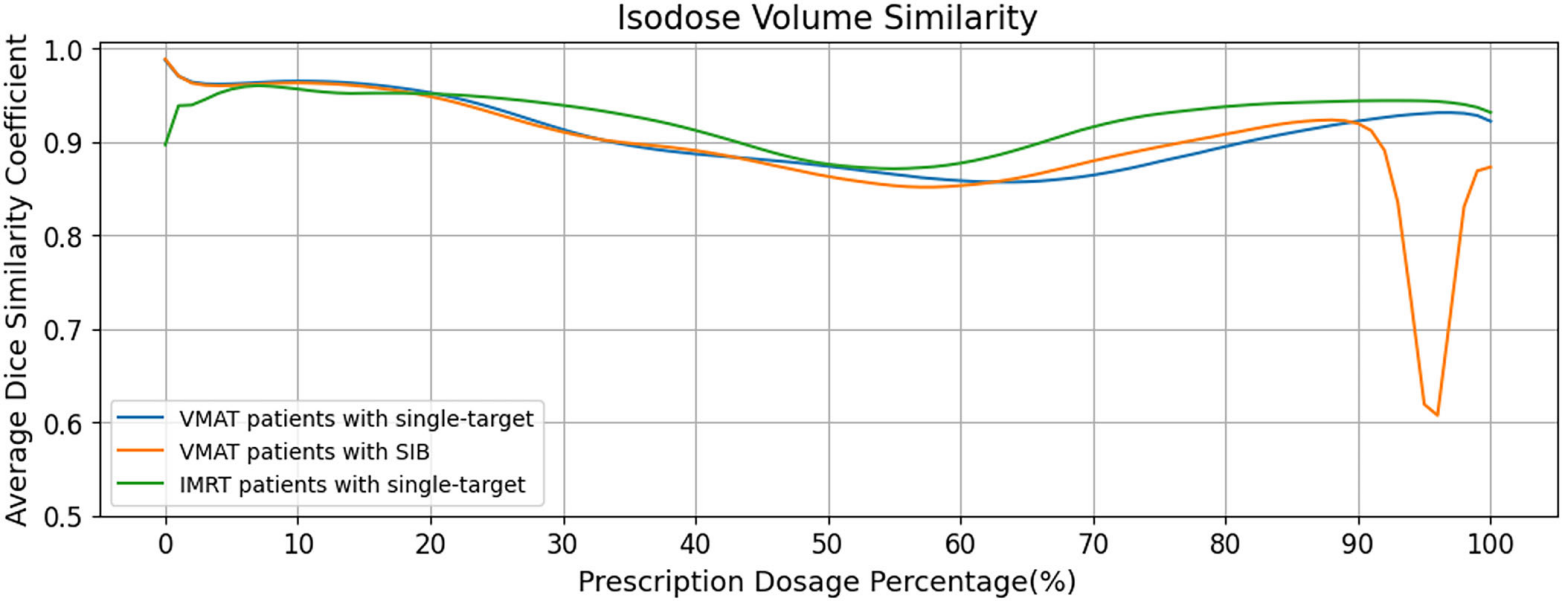
The average dice similarity coefficient for VMAT and IMRT plans with different prescription doses.

Figure 3a,c respectively showed the comparisons among the clinical plan, predicted dose and automatic plan for dose distributions and DVHs for two test patients of VMAT and IMRT. Figure 3b showed the comparisons of the target coverage of prescription isodose lines between the clinical and automatic plan for the same test patients. It can be observed that the automatic plan has a high similarity with the clinical plan in terms of PTV coverage and dose distribution, and was consistent with the distribution trend of the predicted dose. Figure 4 demonstrated the average DSC for VMAT and IMRT plans with different prescription doses. For single‐target plans, the minimum average DSC for VMAT and IMRT patients was 0.86 and 0.87, respectively. For SIB VMAT plans, the DSC curve exhibited a fluctuation (a decrease followed by an increase) within the range of 90%–100% of the prescription dose, i.e., the 45–50 Gy interval, with the minimum DSC value being 0.61, which may result from the sharp escalation of dose within the 45–50 Gy range. Overall, automatic VMAT and IMRT plans achieved better OARs sparing with similar target coverage in comparison with clinically accepted plans.

### 3.4 Planning time assessment

**FIGURE 5:**
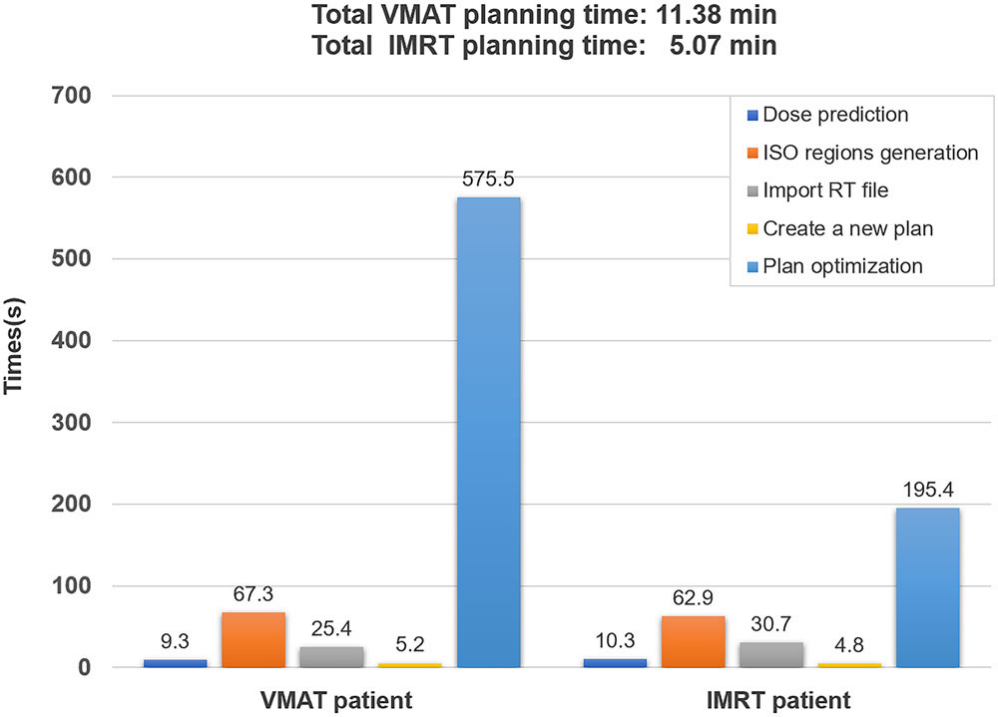
The time required for each step of automatic planning process for randomly selected patients.

The 3D dose distribution predicted from the trained model took approximately 10 s for one patient, and the time required to generate the ISO regions using Python code ranged from 60 to 70 s. The average optimization times for VMAT and IMRT plans were 604.7 and 188.3 s, respectively. Figure 5 showed the time spent in each step of the automatic planning process for a randomly selected VMAT patient and an IMRT patient in the test set. Additionally, in comparison with two dosimetrists with 3 years of experience, the planning time for these two patients were [11.38/25.22] min ([automatic/manual]) and [5.07/17.35] min ([automatic/manual]) for the VMAT and IMRT plans, respectively.

### 3.5 Quality assurance assessment

PSQA was conducted to verify the deliverability of these automatic VMAT and IMRT plans optimized with dose rings and achieved a mean gamma passing rate of 99.1%, 97.1% and 98.3%, and 95.0% under the criteria of 3%/3 mm and 3%/2 mm, respectively. Detailed PSQA results for these VMAT and IMRT plans were presented in Tables S2 and S3, respectively. The high gamma passing rate confirmed the decent deliverability of the automatic plans generated by the method proposed in this study.

## 4 DISCUSSION

In this study, an automatic VMAT and IMRT planning method for generating universally applicable and deliverable RT plans was proposed by integrating DL dose prediction and dose rings optimization. Accurate dose distributions with sufficient target coverage and good OARs sparing were achieved with DL dose prediction models for VMAT and IMRT plans. A better OARs sparing with similar target coverage was achieved using dose rings optimization for automatic VMAT and IMRT plans. High gamma passing rate from 95.0% to 99.1% was achieved during PSQA for these automatically optimized VMAT and IMRT plans with different TPSs. In addition, the automatic plan optimized with dose rings saved more than half of the manual planning time.

Automatic VMAT/IMRT planning usually consists of two steps. One is to predict a high‐quality dose distribution. The second is to transfer this dose distribution into a deliverable plan.^36^ In this study, a F‐ResUnet was adapted to predict the dose distributions for both VMAT and IMRT plans with various dose prescriptions. As shown in Tables 4 and 5, clinically acceptable dose distributions were achieved on PTVs for both VMAT and IMRT plans. Although the predicted dose was a slightly higher on OARs in comparison with clinically accepted plans, no significant difference was observed. This was consistent with the previous study of Yu et al, in which ResUnet and Unet were applied to predict 3D VMAT dose distribution for GC patients with two dose prescriptions.^37^

However, studies pointed out that a DL model achieved a good prediction dose distribution does not guarantee a good deliverable plan when using a commercial TPS to generate the final plan.^38^ DVH based objective functions were usually applied for IMRT/VMAT optimization. However, due to the lack of spatial information, direct prediction of DVH with DL was unfavorable.^14^, ^15^ In this study, dose rings generated after the DL dose prediction were directly converted into as the objective functions for final dose optimization with TPS, which takes the advantage of both accurate 3D dose prediction with sufficient spatial information and simplified final deliverable dose optimization with any TPSs. The method transformed dose distributions of patients into a general format of dose rings, and the constraint functions served the purpose of achieving universality. This implied that all patients predicted by this model could use the same set of constraint functions to generate automated plans of comparable quality to clinical plans. As shown in Figure 3, the dose distribution of automatic IMRT/VMAT plans optimized with dose rings demonstrated a good agreement with those of original clinically accepted plans. Detailed dose metrics evaluation in Tables 4 and 5 also indicated the accuracy of the proposed automatic planning method. The results showed that the HI values of the automated plan are higher than or close to those of the clinical plan, indicating that the dose distribution uniformity within the target area in the automated plan is comparable to that of the clinical plan. Similarly, the CI values of the automated plan are also close to that of the clinical plan, indicating that the dose distribution consistency within the target area is comparable to that of the clinical plan.

**FIGURE 6:**
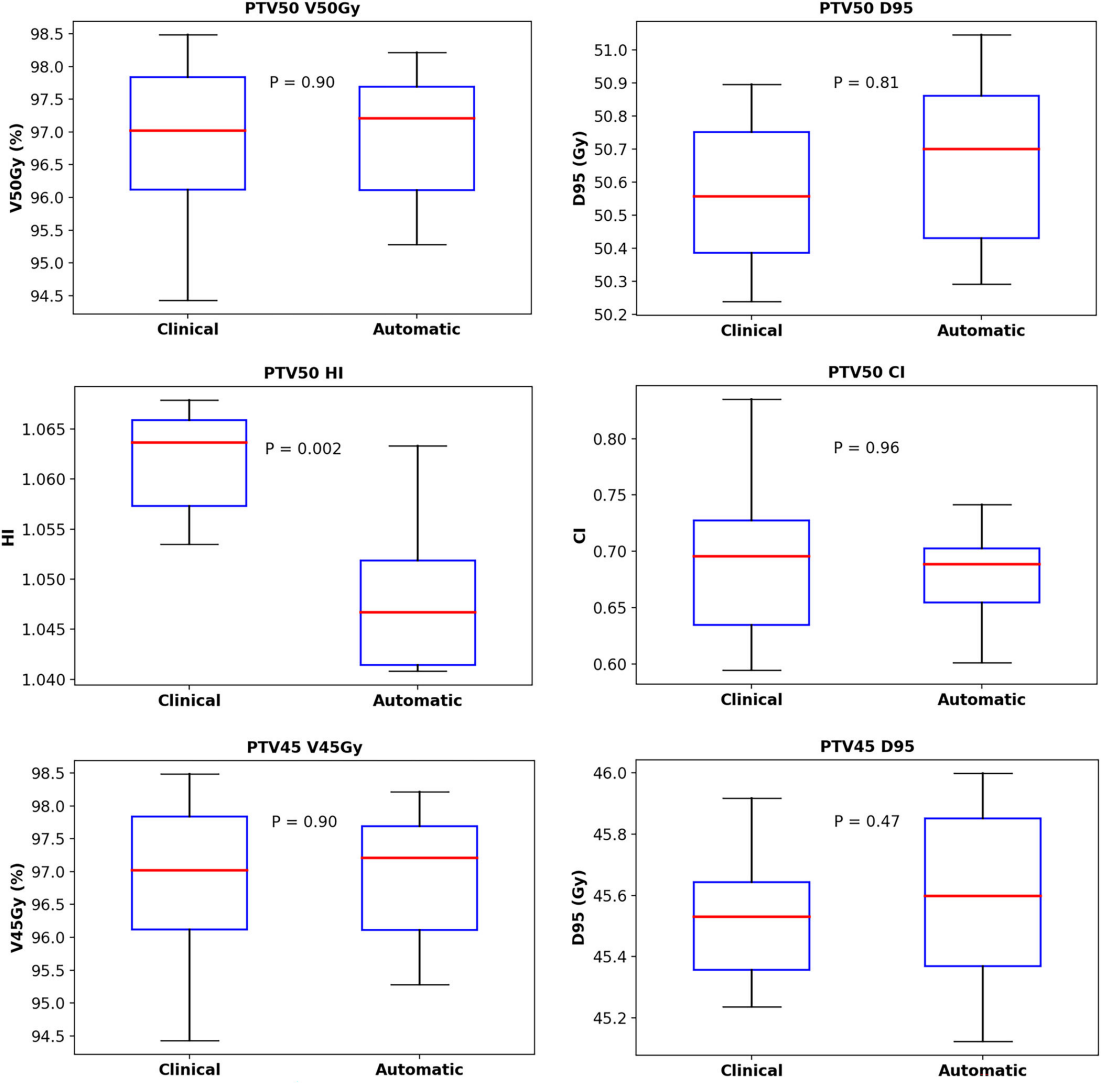
Boxplot comparisons of dosimetric results for PTV45 and PTV50 between the clinical and the automatic plans for SIB patients. Red line represents the median, the blue box represents the quartile, and the top and bottom horizontal black bars represent the maximum and minimum value.

As shown in Figure 4, for single‐target patients in the test set, the minimum average DSCs value was 0.86, indicating good agreement between the dose distribution of automated plans and clinical plans. However, for SIB patients, there was a decrease followed by an increase in average DSC when the dose exceeds 90% of the maximum prescribed dose, which primarily reflects the inherent sensitivity of the DSC to geometric boundary shifts in areas of steep dose gradients.^39^ Even minor spatial misalignments between the dose distribution of automatic plan and the clinical plan within this high‐gradient zone can significantly reduce the DSC value. Critically, this reduction does not necessarily indicate a failure to achieve clinically acceptable absolute dose coverage in the targets. This phenomenon occurs only within the target volumes. Therefore, further analysis was focused on the PTVs of the SIB automatic plans. Figure 6 showed the boxplot comparisons of dosimetric results for PTV45 and PTV50 between the clinical and the automatic plans for SIB patients. Both V50Gy for PTV50 and V45Gy for PTV45 exceeded 95% in all automatic SIB plans, meeting clinical acceptability standards. And there were no significant differences (*p* > 0.05) between clinical and automatic plans for all dose parameters except for the HI of PTV50 (*p* = 0.002). Furthermore, the SIB automatic plans exhibited significantly better homogeneity for PTV50 (with HI closer to 1) compared to the SIB clinical plans. This confirms that despite the DSC fluctuation in the 45–50 Gy interval, the dosimetric quality of target volumes in automatic SIB plans remains comparable to that of clinical plans.

The deliverability of plans generated by the proposed method was further verified with measurement‐based PSQA in this study. As shown in Tables S2 and S3, a mean gamma passing rate of 99.1%, 98.3% and 97.1%, and 95.0% were achieved under criteria of 3%/3 mm and 3%/2 mm for VMAT and IMRT plans optimized with isodose rings, respectively. This outperformed the set passing rate indicated in the TG119 and TG218 for 3%/3 mm and 3%/2 mm criteria.^40^, ^41^ The gamma passing rates of generated VMAT and IMRT plans also surpassed the PSQA results of clinical accepted VMAT (94.7% ± 0.9%) and IMRT (95.2% ± 1.1%) plans at the criteria of 3%/3 mm.^25^ The proposed automatic planning method was able to generate accurate and universally deliverable IMRT and VMAT plans for different TPSs.

One limitation of this study is that a relatively small number of cases was enrolled for DL modeling. Although different IMRT and VMAT plans were trained and tested, cases from other hospitals were needed to further validate the models externally. Another limitation of this study is that it does not consider the spatial distribution of the locations where hotspots occur in the automatic planned dose distribution. Only GC were investigated in this study, automatic planning with this method for more complicated cases, such as head‐and‐neck cancers, should be further investigated.

## 5 CONCLUSIONS

The proposed automatic planning method combining DL dose prediction and dose rings optimization was feasible to generate universally deliverable VMAT and IMRT plans for GC patients using different mainstream TPSs.

## AUTHOR CONTRIBUTIONS

**Weiqian Huang**: Writing—original draft; validation; software; formal analysis; data curation; conceptualization. **Ting Liu**:Validation; data curation; conceptualization. **Yichao Shen**: Software; data curation; supervision; methodology; conceptualization. **Ziqing Xiang**: Resources; data curation. **Dong Wang**: Data curation. **Wen Fu**: Validation. **Li Shao**: Resources. **Xianwen Yu**: Resources. **Weihua Ni**: Data curation. **Yongqiang Zhou**: Methodology. **Huan Liu**: Methodology. **Ce Han**: Methodology. **Xiance Jin**: Writing—review and editing; supervision; funding acquisition; conceptualization. **Ji Zhang**: Validation; investigation; funding acquisition.

## CONFLICT OF INTEREST STATEMENT

The authors declare no conflict of interest.

## Supporting information



Supporting Information

## Data Availability

Research data are stored in an institutional repository and will be shared upon request to the corresponding author.

## References

[1] Chang Y , Yang Z‐Y , Li G‐L , et al. Correlations between radiation dose in bone marrow and hematological toxicity in patients with cervical cancer: a comparison of 3DCRT, IMRT, and RapidARC. Int J Gynecol Cancer. 2016; 26(4): 770‐776. doi:10.1097/igc.0000000000000660 26844613 10.1097/IGC.0000000000000660

[2] Palma D , Vollans E , James K , et al. Volumetric modulated arc therapy for delivery of prostate radiotherapy: comparison with intensity‐modulated radiotherapy and three‐dimensional conformal radiotherapy. Int J Radiat Oncol Biol Phys. 2008; 72(4): 996‐1001. doi:10.1016/j.ijrobp.2008.02.047 18455326 10.1016/j.ijrobp.2008.02.047

[3] Das IJ , Moskvin V , Johnstone PA . Analysis of treatment planning time among systems and planners for intensity‐modulated radiation therapy. J Am Coll Radiol. 2009;6(7):514‐517. doi:10.1016/j.jacr.2008.12.013 19560069 10.1016/j.jacr.2008.12.013

[4] Chanyavanich V , Das SK , Lee WR , Lo JY . Knowledge‐based IMRT treatment planning for prostate cancer. Med Phys. 2011;38(5):2515‐2522. doi:10.1118/1.3574874 21776786 10.1118/1.3574874

[5] Good D , Lo J , Lee WR , Wu QJ , Yin F‐F , Das SK . A Knowledge‐based approach to improving and homogenizing intensity modulated radiation therapy planning quality among treatment centers: an example application to prostate cancer planning. Int J Radiat Oncol Biol Phys. 2013;87(1):176‐181. doi:10.1016/j.ijrobp.2013.03.015 23623460 10.1016/j.ijrobp.2013.03.015

[6] Valdes G , Simone CB , Chen J , et al. Clinical decision support of radiotherapy treatment planning: a data‐driven machine learning strategy for patient‐specific dosimetric decision making. Radiother Oncol. 2017; 125(3): 392‐397. doi:10.1016/j.radonc.2017.10.014 29162279 10.1016/j.radonc.2017.10.014

[7] Delaney AR , Dahele M , Tol JP , Kuijper IT , Slotman BJ , Verbakel WFAR . Using a knowledge‐based planning solution to select patients for proton therapy. Radiother Oncol. 2017;124(2):263‐270. doi:10.1016/j.radonc.2017.03.020 28411963 10.1016/j.radonc.2017.03.020

[8] Chang ATY , Hung AWM , Cheung FWK , et al. Comparison of planning quality and efficiency between conventional and knowledge‐based algorithms in nasopharyngeal cancer patients using intensity modulated radiation therapy. Int J Radiat Oncol Biol Phys. 2016; 95(3): 981‐990. doi:10.1016/j.ijrobp.2016.02.017 27302513 10.1016/j.ijrobp.2016.02.017

[9] Yuan L , Wu QJ , Yin FF , Jiang Y , Yoo D , Ge Y . Incorporating single‐side sparing in models for predicting parotid dose sparing in head and neck IMRT. Med Phys. 2014;41(2):021728. doi:10.1118/1.4862075 24506619 10.1118/1.4862075 PMC3977781

[10] Shiraishi S , Tan J , Olsen LA , Moore KL . Knowledge‐based prediction of plan quality metrics in intracranial stereotactic radiosurgery. Med Phys. 2015;42(2):908‐917. doi:10.1118/1.4906183 25652503 10.1118/1.4906183

[11] Nguyen D , Jia X , Sher D , et al. 3D radiotherapy dose prediction on head and neck cancer patients with a hierarchically densely connected U‐net deep learning architecture. Phys Med Biol. 2019; 64(6): 065020. doi:10.1088/1361‐6560/ab039b 30703760 10.1088/1361-6560/ab039b

[12] Nguyen D , Long T , Jia X , et al. A feasibility study for predicting optimal radiation therapy dose distributions of prostate cancer patients from patient anatomy using deep learning. Sci Rep. 2019; 9(1): 1076. doi:10.1038/s41598‐018‐37741‐x 30705354 10.1038/s41598-018-37741-x PMC6355802

[13] Gronberg MP , Gay SS , Netherton TJ , Rhee DJ , Court LE , Cardenas CE . Technical note: dose prediction for head and neck radiotherapy using a three‐dimensional dense dilated U‐net architecture. Med Phys. 2021;48(9):5567‐5573. doi:10.1002/mp.14827 34157138 10.1002/mp.14827

[14] Wang C , Zhu X , Hong JC , Zheng D . Artificial intelligence in radiotherapy treatment planning: present and future. Technol Cancer Res Treat. 2019;18:1533033819873922. doi:10.1177/1533033819873922 31495281 10.1177/1533033819873922 PMC6732844

[15] McIntosh C , Welch M , McNiven A , Jaffray DA , Purdie TG . Fully automated treatment planning for head and neck radiotherapy using a voxel‐based dose prediction and dose mimicking method. Phys Med Biol. 2017;62(15):5926‐5944. doi:10.1088/1361‐6560/aa71f8 28486217 10.1088/1361-6560/aa71f8

[16] Kearney V , Chan JW , Haaf S , Descovich M , Solberg TD . DoseNet: a volumetric dose prediction algorithm using 3D fully‐convolutional neural networks. Phys Med Biol. 2018;63(23):235022. doi:10.1088/1361‐6560/aaef74 30511663 10.1088/1361-6560/aaef74

[17] Chen X , Men K , Li Y , Yi J , Dai J . A feasibility study on an automated method to generate patient‐specific dose distributions for radiotherapy using deep learning. Med Phys. 2018;46(1):56‐64. doi:10.1002/mp.13262 30367492 10.1002/mp.13262 PMC7379709

[18] Fan J , Wang J , Chen Z , Hu C , Zhang Z , Hu W . Automatic treatment planning based on three‐dimensional dose distribution predicted from deep learning technique. Med Phys. 2018;46(1):370‐381. doi:10.1002/mp.13271 30383300 10.1002/mp.13271

[19] Shen Y , Tang X , Lin S , Jin X , Ding J , Shao M . Automatic dose prediction using deep learning and plan optimization with finite‐element control for intensity modulated radiation therapy. Med Phys. 2023;51(1):545‐555. doi:10.1002/mp.16743 37748133 10.1002/mp.16743

[20] Zhong Y , Yu L , Zhao J , et al. Clinical implementation of automated treatment planning for rectum intensity‐modulated radiotherapy using voxel‐based dose prediction and post‐optimization strategies. Front Oncol. 2021; 11: 697995. doi:10.3389/fonc.2021.697995 34249757 10.3389/fonc.2021.697995 PMC8264432

[21] Sun Z , Xia X , Fan J , et al. A hybrid optimization strategy for deliverable intensity‐modulated radiotherapy plan generation using deep learning‐based dose prediction. Med Phys. 2022; 49(3): 1344‐1356. doi:10.1002/mp.15462 35043971 10.1002/mp.15462

[22] Heilemann G , Zimmermann L , Schotola R , et al. Generating deliverable DICOM RT treatment plans for prostate VMAT by predicting MLC motion sequences with an encoder‐decoder network. Med Phys. 2023; 50(8): 5088‐5094. doi:10.1002/mp.16545 37314944 10.1002/mp.16545

[23] Yuan Z , Wang Y , Hu P , et al. Accelerate treatment planning process using deep learning generated fluence maps for cervical cancer radiation therapy. Med Phys. 2022; 49(4): 2631‐2641. doi:10.1002/mp.15530 35157337 10.1002/mp.15530

[24] Ma L , Chen M , Gu X , Lu W . Deep learning‐based inverse mapping for fluence map prediction. Phys Med Biol. 2020;65(23):235035. doi:10.1088/1361‐6560/abc12c 10.1088/1361-6560/abc12c PMC8044255 33053515

[25] Mason D . SU‐E‐T‐33: pydicom: an open source DICOM Library. Med Phys. 2011;38(6Part10):3493‐3493. doi:10.1118/1.3611983

[26] van der WaltS , Colbert SC , Varoquaux G . The NumPy array: a structure for efficient numerical computation. Comput Sci Eng. 2011;13(2):22‐30. doi:10.1109/mcse.2011.37

[27] Babier A , Zhang B , Mahmood R , et al. OpenKBP: the open‐access knowledge‐based planning grand challenge and dataset. Med Phys. 2021; 48(9): 5549‐5561. doi:10.1002/mp.14845 34156719 10.1002/mp.14845

[28] Hernandez V , Hansen CR , Widesott L , et al. What is plan quality in radiotherapy? The importance of evaluating dose metrics, complexity, and robustness of treatment plans. Radiother Oncol. 2020; 153: 26‐33. doi:10.1016/j.radonc.2020.09.038 32987045 10.1016/j.radonc.2020.09.038

[29] Ronneberger O , Fischer P , Brox T . U‐net: convolutional networks for biomedical image segmentation. in: Lecture Notes in Computer Science (Including Subseries Lecture Notes in Artificial Intelligence and Lecture Notes in Bioinformatics). Springer Verlag, pp. 234‐241. doi:10.1007/978‐3‐319‐24574‐4_28

[30] Wang J , Hu J , Song Y , et al. VMAT dose prediction in radiotherapy by using progressive refinement UNet. Neurocomputing. 2022; 488: 528‐539. doi:10.1016/j.neucom.2021.11.061

[31] Yi J , Han C , Zheng X , et al. Individual volume‐based 3D gamma indices for pretreatment VMAT QA. J Appl Clin Med Phys. 2017; 18(3): 28‐36. doi:10.1002/acm2.12062 28318101 10.1002/acm2.12062 PMC5689866

[32] Jin X , Yan H , Han C , Zhou Y , Yi J , Xie C . Correlation between gamma index passing rate and clinical dosimetric difference for pre‐treatment 2D and 3D volumetric modulated arc therapy dosimetric verification. Br J Radiol. 2015;88(1047):20140577. doi:10.1259/bjr.20140577 25494412 10.1259/bjr.20140577 PMC4651188

[33] Hodapp N . Der ICRU‐Report 83: verordnung, Dokumentation und Kommunikation der fluenzmodulierten Photonenstrahlentherapie (IMRT). Strahlenther Onkol. 2012;188(1):97‐100. doi:10.1007/s00066‐011‐0015‐x 22234506 10.1007/s00066-011-0015-x

[34] Avt Riet , Mak ACA , Moerland MA , Elders LH , van der Zee W . A conformation number to quantify the degree of conformality in brachytherapy and external beam irradiation: application to the prostate. Int J Radiat Oncol Biol Phys. 1997;37(3):731‐736. doi:10.1016/s0360‐3016(96)00601‐3 9112473 10.1016/s0360-3016(96)00601-3

[35] Tanabe Y , Ishida T , Eto H , Sera T , Emoto Y . Evaluation of the correlation between prostatic displacement and rectal deformation using the Dice similarity coefficient of the rectum. Med Dosim. 2019;44(4):e39‐e43. doi:10.1016/j.meddos.2018.12.005 30642696 10.1016/j.meddos.2018.12.005

[36] Zimmermann L , Faustmann E , Ramsl C , Georg D , Heilemann G . Technical note: dose prediction for radiation therapy using feature‐based losses and one cycle learning. Med Phys. 2021;48(9):5562‐5566. doi:10.1002/mp.14774 34156727 10.1002/mp.14774 PMC8518421

[37] Yu W , Xiao C , Xu J , Jin J , Jin X , Shen L . Direct dose prediction with deep learning for postoperative cervical cancer underwent volumetric modulated arc therapy. Technol Cancer Res Treat. 2023;22:15330338231167039. doi:10.1177/15330338231167039 36999201 10.1177/15330338231167039 PMC10071211

[38] Babier A , Mahmood R , McNiven AL , Diamant A , Chan TCY . The importance of evaluating the complete automated knowledge‐based planning pipeline. Physica Med. 2020;72:73‐79. doi:10.1016/j.ejmp.2020.03.016 10.1016/j.ejmp.2020.03.016 32222642

[39] Duan Y , Wang J , Wu P , et al. AS‐NeSt: a novel 3D deep learning model for radiation therapy dose distribution prediction in esophageal cancer treatment with multiple prescriptions. Int J Radiat Oncol Biol Phys. 2024; 119(3): 978‐989. doi:10.1016/j.ijrobp.2023.12.001 38159780 10.1016/j.ijrobp.2023.12.001

[40] Li H , Dong L , Zhang L , Yang JN , Gillin MT , Zhu XR . Toward a better understanding of the gamma index: investigation of parameters with a surface‐based distance methoda). Med Phys. 2011;38(12):6730‐6741. doi:10.1118/1.3659707 22149855 10.1118/1.3659707 PMC3298565

[41] Ezzell GA , Burmeister JW , Dogan N , et al. IMRT commissioning: multiple institution planning and dosimetry comparisons, a report from AAPM Task Group 119. Med Phys. 2009; 36(11): 5359‐5373. doi:10.1118/1.3238104 19994544 10.1118/1.3238104

