# Supplementary material for: Automatic radiotherapy planning for deliverable plans using deep learning dose prediction and dose rings optimization in cervical cancer
Source: J Appl Clin Med Phys. 2025 Nov 12;26(11):e70353. doi: 10.1002/acm2.70353 (PMC12611595; doi:10.1002/acm2.70353)
Supplement: Supplementary file 1 — Supporting Information [file ACM2-26-e70353-s001.docx]

**supplementary file one**

*Dose distribution prediction*

For dose prediction, our data comprised 3D stacks of CT scans, structure masks, and RD dose information. Due to GPU memory limitations, we segmented the complete data stacks (512×512× number of slices) into smaller segments of 16 slices each, starting from both the first and last slices of the entire dataset. Subsequently, PyTorch was utilized to resize these segmented stacks to a single-slice resolution of 256×256.

For VMAT model training, mean squared error (MSE), mean absolute error (MAE) and Rank loss were applied for the model training, which is formulated as Equation (1):

Loss = MSE + 0.5MAE + 0.1Rank (1)

The Rank loss measures the difference between the predicted dose and the actual dose by considering the relative ranking of the predicted and actual dose voxel according to scale rather than the absolute values. The aim of adding Rank loss is to minimize this difference in ranking, thereby improving the accuracy of dose prediction. For IMRT model training, only MSE and MAE loss functions were used.

The neural network was trained with randomly initialized weights. We employed the Adam optimizer to update parameters for each training sample. Considering the memory requirements of deeper architectures on the GPU VRAM, we set the batch size to four based on the network’s layer depth. A learning rate of 0.0001 was used for training, with a decay applied to it. The final model was chosen at the training epoch when the loss on the validation set stopped decreasing which guarantees that model stopped learning image features and prevented overfitting on the training data.

To assess the performance and stability of the developed F-ResUNet model, it was compared with 3 classical 3D CNN network models commonly used for dose prediction: Residual U-Net (ResUNet), Hierarchically Densely Connected U-Net (HD-UNet),^1^ and Cascade 3D U-Net (C3D-UNet).^2^ Table S1 showed the comparative results between the proposed F-ResUNet model and three other models. It can be observed that our model generally exhibits the lowest average absolute error across various dose metrics, especially in the OARs region.

1 Nguyen D, Jia X, Sher D, et al. 3D radiotherapy dose prediction on head and neck cancer patients with a hierarchically densely connected U-net deep learning architecture. Phys Med Biol. 2019;64(6):065020.

2 Liu S, Zhang J, Li T, Yan H, Liu J. Technical Note: A cascade 3D U-Net for dose prediction in radiotherapy. Med Phys. 2021;48(9):5574-5582.

**supplementary file two**

***Evaluation metrics***

The evaluation metrics of OARs included bladder (V_45Gy_, V_20Gy_, and D_mean_), rectum (V_45Gy_, V_20Gy_, and D_mean_), the left and right femoral head (V_30Gy_ and D_mean_), small intestine (V_40Gy_, V_30Gy_, and D_mean_). V_n_ represents the volume percentage of the corresponding OAR that received at least n Gy. PTV (V_45Gy_, V_50Gy_ and D_95_), homogeneity index (HI), and conformity index (CI) were used to assess the PTV coverage, which were defined as:

HI = D_5_ ∕ D_95_ (1)

CI = (V_PTV_ ∩ V_100%_) ^2^ ∕ (V_PTV_ × V_100%_) (2)

where D_x_ denotes the corresponding dose of X% volume of the target. V_PTV_ is the volume of the PTV, and V_100%_ is the volume encompassed by the 100% isodose line.

In addition, DSCs of various isodose volumes between the clinical plans and automatic plans were also calculated to evaluate the dose distribution similarity, which is defined as:

DSC = 2 (A ∩ B) ∕ (A + B) (3)

where A represents predicted isodose volumes and B represents clinical isodose volumes.

**Supplementary Tables**

**Table S1. Mean absolute differences in the dose metrics between prediction and manual plans with standard deviations for test samples in the four tested model cases.**

| **Organs** | **Dose metrics** | **ResUNet** | **HD U-net** | **C3D U-net** | **F-ResUNet(Ours)** |
| --- | --- | --- | --- | --- | --- |
| PTV50 | V_50Gy_(%) | 1.95 ± 1.20 | **1.36 ± 0.95** | 2.05 ± 1.58 | 1.77 ± 1.36 |
|  | D_95_(Gy) | 0.58 ± 0.29 | 0.54 ± 0.42 | 0.58 ± 0.32 | **0.53 ± 0.29** |
| PTV45 | V_45Gy_(%) | 1.65 ± 1.11 | 1.92 ± 1.16 | **1.38 ± 0.99** | 1.88 ± 1.15 |
|  | D_95_(Gy) | 0.45 ± 0.30 | 0.39 ± 0.28 | **0.30 ± 0.23** | 0.67 ± 0.35 |
|  | CI | 0.03 ± 0.02 | 0.03 ± 0.02 | 0.04 ± 0.02 | **0.02 ± 0.01** |
|  | HI | **0.01 ± 0.01** | **0.01 ± 0.01** | 0.02 ± 0.01 | 0.03 ± 0.01 |
| Bladder | D_mean_ (Gy) | 1.97 ± 2.14 | 1.75 ± 1.72 | 1.89 ± 1.88 | **1.73 ± 1.60** |
|  | V_45Gy_(%) | 3.46 ± 5.12 | 2.94 ± 3.70 | 3.17 ± 4.24 | **2.76 ± 2.65** |
|  | V_20Gy_(%) | 7.17 ± 7.65 | 6.92 ± 6.04 | 6.43 ± 7.18 | **6.29 ± 6.38** |
| Rectum | D_mean_(Gy) | 1.94 ± 1.52 | 2.03 ± 1.44 | 1.67 ± 1.35 | **1.55 ± 1.34** |
|  | V_45Gy_(%) | 6.97 ± 5.34 | 7.67 ± 4.18 | 6.83 ± 3.39 | **6.51 ± 4.06** |
|  | V_20Gy_(%) | 2.82 ± 3.74 | 2.86 ± 3.26 | 2.48 ± 3.06 | **2.01 ± 3.02** |
| L-femoral head | D_mean_(Gy) | 0.87 ± 0.58 | 0.88 ± 0.55 | 0.94 ± 0.47 | **0.83 ± 0.50** |
|  | V_30Gy_(%) | 3.20 ± 2.82 | **2.84 ± 1.85** | 2.86 ± 2.17 | 3.15 ± 2.49 |
| R-femoral head | D_mean_(Gy) | 1.25 ± 1.09 | 1.29 ± 1.09 | 1.31 ± 1.11 | **1.20 ± 0.96** |
|  | V_30Gy_(%) | 4.36 ± 4.27 | 4.23 ± 3.92 | 4.58 ± 4.12 | **3.82 ± 4.10** |
| Small intestine | D_mean_(Gy) | 0.40 ± 0.33 | 0.41 ± 0.33 | 0.40 ± 0.30 | **0.38 ± 0.44** |
|  | V_40Gy_(%) | 0.96 ± 1.10 | **0.95 ± 1.18** | 1.01 ± 1.16 | 0.96 ± 1.39 |
|  | V_30Gy_(%) | 1.43 ± 1.19 | 1.27 ± 1.08 | 1.46 ± 1.15 | **1.16 ± 1.42** |

Note: Mean values and standard deviations are shown. Bold font indicates the minimum mean absolute error under the same dose metric.

**Table S2 Gamma passing rates for 22 VMAT automated plans.**

| **Patient** | **3%/3mm** | **3%/2mm** |
| --- | --- | --- |
| #1 | 98.7% | 95.7% |
| #2 | 99.7% | 97.8% |
| #3 | 98.4% | 95.2% |
| #4 | 99.2% | 97.4% |
| #5 | 99.3% | 97.3% |
| #6 | 99.3% | 97.7% |
| #7 | 98.9% | 96.8% |
| #8 | 99.3% | 97.6% |
| #9 | 99.2% | 97.7% |
| #10 | 98.7% | 96.7% |
| #11 | 99.3% | 97.9% |
| #12 | 98.9% | 96.6% |
| #13 | 99.2% | 97.6% |
| #14 | 98.2% | 94.9% |
| #15 | 98.6% | 96.1% |
| #16 | 99.5% | 99.0% |
| #17 | 99.7% | 97.7% |
| #18 | 98.9% | 96.7% |
| #19 | 99.9% | 99.0% |
| #20 | 99.3% | 97.6% |
| #21 | 99.1% | 97.1% |
| #22 | 98.8% | 96.5% |
| **Mean±SD** | **99.1±0.4%** | **97.1±1.0%** |

**Table S3 Gamma passing rates for 10 IMRT automated plans.**

| **Patient** | **3%/3mm** | **3%/2mm** |
| --- | --- | --- |
| #1 | 98.9% | 96.1% |
| #2 | 97.5% | 93.9% |
| #3 | 97.1% | 94.8% |
| #4 | 97.4% | 93.8% |
| #5 | 97.3% | 93.6% |
| #6 | 98.5% | 94.6% |
| #7 | 99.8% | 97.1% |
| #8 | 99.0% | 96.0% |
| #9 | 98.2% | 94.4% |
| #10 | 98.9% | 95.7% |
| **Mean±SD** | **98.3±0.9%** | **95.0±1.2%** |
